# Arginine deprivation, growth inhibition and tumour cell death: 3. Deficient utilisation of citrulline by malignant cells

**DOI:** 10.1038/sj.bjc.6601134

**Published:** 2003-07-29

**Authors:** D N Wheatley, E Campbell

**Affiliations:** 1 Department of Cell Pathology, University of Aberdeen, MacRobert Building, 581 King Street, Aberdeen AB24 5UA, UK

**Keywords:** citrulline, metabolism, deficiency, arginine deprivation, melanoma

## Abstract

Arginine deprivation causes death of up to 80% of cancer cell lines *in vitro*, but in the body, citrulline would be available as a convertible source of this amino acid *in vivo*. Some tumour cell lines, notably the vast majority of melanomas and hepatocellular carcinomas, tend to be deficient in argininosuccinate synthetase (EC 6.5.4.3.), and therefore cannot recycle citrulline to arginine. Argininosuccinate synthetase is present at levels that convert enough citrulline to arginine to allow limited growth in about half of a modest range of malignant cell types analysed in this study. Attempts to rescue cells that are unable to utilise citrulline with the immediate downstream product, argininosuccinate, had very limited success in a few tumour cell lines. Particularly noteworthy is the demonstration that argininosuccinate was totally incapable of rescuing cells that utilise citrulline efficiently, consistent with tight channelling (coupling) of argininosuccinate synthetase and argininosuccinate lyase in the urea cycle. The findings suggest that an excellent opportunity exists for further exploitation of arginine deprivation in the selective killing of tumour cells.

Arginine is a vital amino acid for many metabolic processes other than protein synthesis (e.g. creatine production, polyamine synthesis and nitric oxide (NO) generation). Its removal from culture medium by medium formulation or arginase treatment quickly leads to death in ∼80% of tumour cell lines (Scott *et al*, 2000). The addition of citrulline can often circumvent this deficiency, but few cells are capable of its biosynthesis in culture. Arginine deficiency *in vivo* has been induced by various means, but is less effectively achieved because body homeostasis is too robust, and citrulline generation is difficult to control. Having successfully reduced L1210 cells in culture to negligible viability in 3–5 days with arginase (EC 3.5.3.1), citrulline supplementation in the continued presence of the enzyme partially circumvented the arginine deficiency. The rate of conversion became a rate-limiting factor, most noticeably in such fast-growing leukaemia as L1210 (Philip *et al*, 2003). In a corresponding *in vivo* study, arginase treatment of DBA/2 mice injected intraperitoneally with 1210 cells increased neither their survival time nor decreased tumour burden, despite plasma arginine falling to ∼1–2 *μ*M level. Plasma citrulline remained normal, and therefore the tumour cells must have converted citrulline to arginine fast enough to sustain relatively normal tumour growth rate (Wu and Morris, 1998; Philip *et al*, 2003). This makes it difficult to understand how previous *in vivo* work with arginase and arginine deiminase could have achieved significant inhibition of tumour growth under similar conditions (Bach and Swaine, 1965; Miyazaki *et al*, 1990), and begs further analysis of the ability of tumour cells to use citrulline *in lieu* of arginine (Wheatley and Campbell, 2002). Sugimura *et al* (1992) found that four out of five human melanoma cell lines deprived of arginine with arginine deiminase (EC 3.5.3.6) could not be rescued with citrulline, but grew when argininosuccinate, the immediate downstream intermediate, was supplied. Another tumour type that is characteristically deficient in this enzyme is human hepatocellular carcinoma (Ensor *et al*, 2002). With regard to melanomas, however, we found that B16F10, a murine melanoma cell line, was not deficient in argininosuccinate synthetase, and therefore an inability to handle citrulline is *not* an idiosyncratic feature of melanomas, at least not in the mouse. Since B16F10 cells grew well on citrulline with little curtailment of its kinetics, we carefully selected a small panel of other tumour and transformed cell lines, mostly of human origin, for a preliminary *in vitro* analysis, which included several examples of melanomas, leukaemias and osteosarcomas. The primary aim was to establish the degree of variability in the metabolism of citrulline in cultured cells *before* embarking on the more difficult and extensive task of analysing tumour biopsies and primary cultures grown from them.

## MATERIALS AND METHODS

### Cell culture

Cells from a wide range of suppliers and sources were grown in RPMI 1640 medium (Life Technologies, Paisley, UK) at 37°C in a humidified atmosphere of 5% CO_2_. Cell counting involved a Coulter electronic particle counter (Model ZM; Coulter Electronics, Luton). Counts are representative of the normal cell population, within standard parameters, and unless otherwise stated cannot be taken as an indication of viability. All cell cultures were routinely screened, and just prior to experimental use, for four types of mycoplasma, using the Mycoplasma Screening Kit of Roche (Mannheim, Germany), according to the manufacturer's instructions to ensure that no cultures used for experimental work tested positive.

### Experimental design

Cells were seeded at approximately 50 000 cells per well in 12-well plates and allowed to settle in RPMI medium containing 1 mM arginine and 5% dialysed foetal calf serum (FCS) overnight. Prior to treatment, cells were washed with prewarmed arginine-free medium (hereafter AFM) and treated with fresh AFM to which arginine, argininosuccinate, citrulline and ornithine was added at 1 mM, where appropriate. Native bovine arginase (Sigma, Poole, UK) or human recombinant arginase (Kyoto, Japan) was added at a concentration of 1 U ml^−1^ to arginine-containing RPMI. In the case of L1210 cells, a suspension culture line, cells were treated immediately on being resuspended at the desired concentration in AFM to which arginine, argininosuccinate, citrulline and ornithine were added as desired.

### Enzyme activity

Arginase was analysed *in vitro* overtime at 37°C in a humidified atmosphere of 5% CO_2_ to show that it maintained activity over the course of each experiment, using the ornithine method of Ochoa *et al* (2000). The specific activity of arginase was given as the amount of ornithine produced in *μ*mol min^−1^ mg protein^−1^. In all, 1 U of arginase was taken as the amount that broke down 1 *μ*mol arginine min^−1^.

## RESULTS AND DISCUSSION

### Cell differences in citrulline and argininosuccinate utilisation

#### Melanomas

Table 1

**Table 1:** Summary of the ability of a range of human and murine normal and malignant cell lines to utilise citrulline, argininosuccinate and arginine

					**Type C**	
**Cell designation**	**Species**	**Origin**	**Type A**^a^	**Type B**	**AS+**	**AS−**
JMF9	Human	Skin fibroblast				+
						
A375	Human	Melanoma		+		
MEWO	Human	Melanoma		+		
G361	Human	Melanoma	+			
B16F10^b^	Murine	Melanoma				+
						
HL428	Human	Leukaemia				+
JURKAT	Human	Leukaemia				+
L1210	Murine	Leukaemia				+
						
HeLa	Human	Cervical carcinoma				+
SAos-2	Human	Osteogenic sarcoma		+		
CHO	Chinese hamster	Ovary	+			

a This refers to the absence of growth when twice the concentration of citrulline was added instead of arginine.

b Non-human lines shaded. Type A: grow only when arginine is present; Type B: grow on arginine or argininosuccinate, but not citrulline; Type C/As+: grow on citrulline *and* argininosuccinate; Type C/As−: grow on citrulline, but cannot grow on argininosuccinate.

is a compilation of our findings on 11 malignant or transformed cell lines and one normal control (human diploid fibroblasts, designated JMF9). Fibroblasts have the ability to use citrulline, and their behaviour is consistent with tight coupling of the citrulline → arginine pathway (Cheung *et al*, 1989; Schimke, 1964) because of their complete inability to handle argininosuccinate in the absence of citrulline. The melanoma data in Table 1 are in accord with Sugimura *et al* (1992) findings in melanomas; our data confirm that two of them were unable to metabolise citrulline (A375 and MEWO), but that they could grow in argininosuccinate-supplemented medium. The third melanoma line, G361, could survive in citrulline but showed little or no real growth, indicating a very low maintenance level of argininosuccinate synthetase. It therefore has almost total dependence on arginine, also in accord with the data of Sugimura *et al* (1992).

The findings on human melanomas contrast sharply with those on mouse B16F10 melanoma cells, which efficiently handle citrulline (Table 1), but unlike A375 and MEWO, showed no signs of being rescued by argininosuccinate. Therefore, clear distinctions exist between melanoma cell lines in their ability to handle citrulline and argininosuccinate.

Ensor *et al* (2002) found a complete absence of argininosuccinate synthetase in both human melanoma and hepatocellular carcinoma cell lines. The fact that close to half of *all* tumour cell types given in Table 1 failed to convert citrulline seems a high percentage to us, even when we know that these other investigators confined themselves to melanomas and hepatocellular carcinomas (Sugimura *et al*, 1992; Ensor *et al*, 2002). However, the latter authors do concede that several different tumour cells types are not inhibited so strongly by arginine deprivation and can use citrulline, although they furnished no actual data on them other than their designations.

#### Other tumour types

With regard to osteosarcoma, SAos-2 behaved in the same manner as G361, which is Type A. Another osteosarcoma, SAos-2, behaved in a similar manner to MEWO in utilising argininosuccinate, but not citrulline, and is therefore Type B. This again is consistent with tight metabolic channelling, excluding argininosuccinate from the pathway when it is fully intact, and ensuring that only endogenous argininosuccinate (that generated *in situ* from citrulline) is used by the lyase to form arginine. The three human leukaemic cell lines (L1210, Jurkat and HL428, see Table 1) had normal phenotypes with regard to arginine and citrulline metabolism. Although Sugimura *et al* (1990) showed that some leukaemic cell lines may overexpress argininosuccinate synthetase, we found no evidence that citrulline was the preferred substrate. Less is known about leukaemic cultures with respect to the relevant enzymes of the urea cycle, and clearly a more extensive series of analyses is required before we can establish whether the relationship with mRNA copy number per cell for argininosuccinate synthetase holds fast (work in progress) as in melanomas and hepatocellular carcinomas. However, the point to be emphasised is that leukaemias are diametrically opposite to (human) melanoma or hepatocellular carcinomas with regard to arginine → citrulline metabolism.

#### Contamination of argininosuccinate with arginine

**Figure 1:**
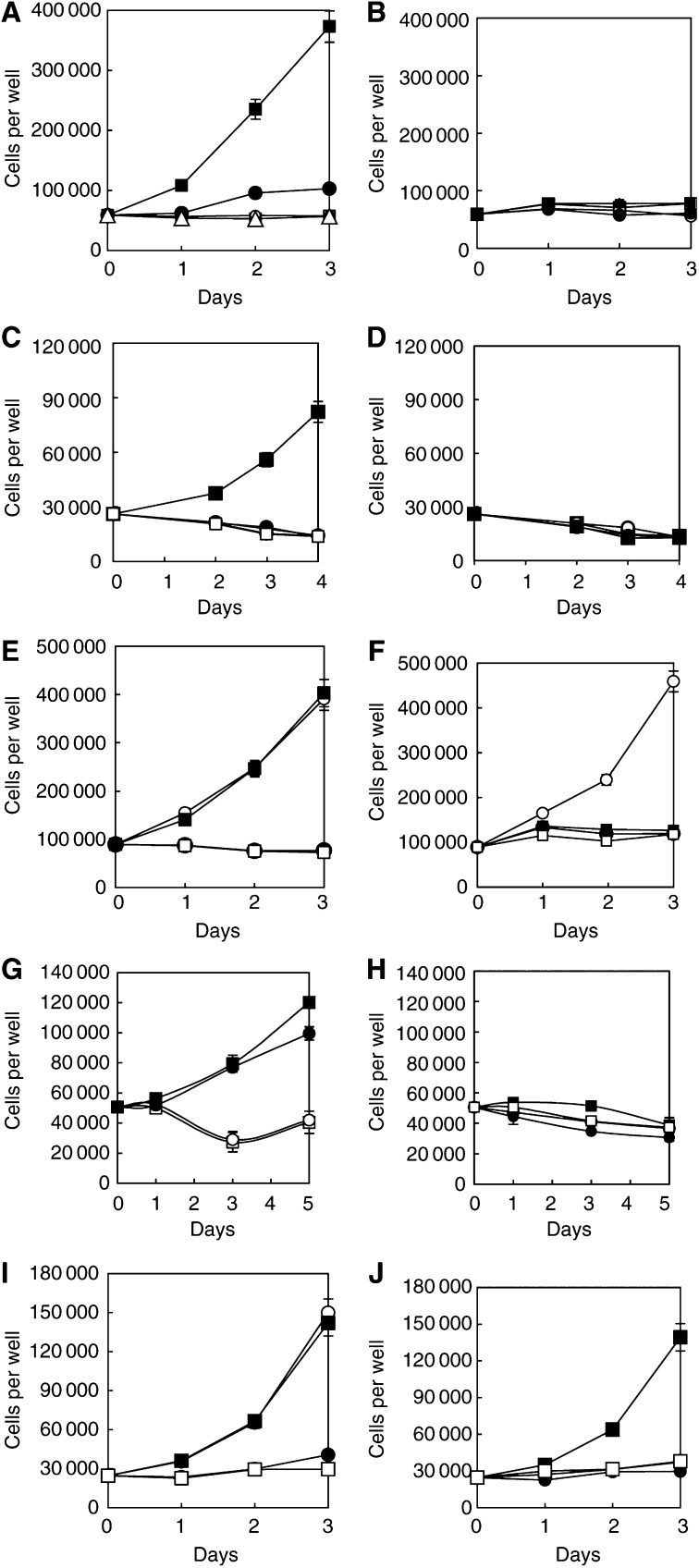
The left-hand side of each pair is made arginine-deficient in RPMI 1640 during preparation and has a 5% dialysed serum supplement. The right-hand side of each pair refers to cultures in complete RPMI 1640 with 5% dialysed serum, but to which was added 1 U ml^−1^ human recombinant arginase. The symbols are the same throughout: □, no supplementation (arginine deficient control); ▪, arginine (1 mM); ○, citrulline (1 mM); •, argininosuccinate (1 mM); ▵, ornithine (1 mM; Figure 1 only). The points are means of three determinations, with the bars (often hidden within the symbols) giving 1 s.d. of the mean. Each cell line was the subject of more than two experiments from which these data were graphed, and most have been examined many more times since these data were first compiled, with similar results. (**A**, **B**) A375; (**C**, **D**) CHO; (**E**, **F**) B16F10; (**G**, **H**) MEWO; (**I**, **J**) L1210.

Depending on how argininosuccinate was prepared, the product could have free arginine as a contaminant. This possibility was eliminated by HPLC analysis of the product, which gave no significant arginine peak. Breakdown soon after the preparation of argininosuccinate medium did not show any released (free) arginine. Although rescue with argininsuccinate in AFM with traces of contaminating arginine is therefore very unlikely, it would have been pre-emptorily destroyed before the cells could make use of it where arginase had been used to create the deficiency (see Figure 1 a–j

).

## CONCLUDING REMARKS

The small sample of cell lines examined in our study shows that half are deficient in argininosuccinate synthetase, with melanomas being well represented. The data on argininosuccinate usage in relation to citrulline convincingly illustrate that a tightly coupled pathway (or channel) normally operates, thereby preventing the access of argininosuccinate to argininosuccinate lyase (EC 4.3.2.1.), which would explain the absence of Type C/AS^+^ tumours (Table 1). This pathway seems to be disrupted, or at least much less tight, in other cell lines of malignant and transformed phenotypes, but so far has not applied to any of the leukaemic lines analysed.

Rescue of cell lines incapable of utilising citrulline with argininosuccinate does occur, but with considerable differences between the cell lines. CHO is an excellent example of a cell line that cannot use it and is therefore totally dependent on arginine. This is more noteworthy because the generalisation of Eagle (1959) based on a couple of transformed lines (mouse L strain cells) and malignant lines (HeLa and KB) that citrulline can substitute for arginine is frankly not true across the board (cf also Schimke, 1964). However, MEWO can use argininosuccinate, and A375 cells grow well on it, whereas G361 survive although they do not proliferate to any significant extent in its presence. We should also note that normal fibroblasts manage well on citrulline, but do not thrive in the presence of argininosuccinate (Table 1; Philip *et al*, 2003). However, until more extensive studies are carried out we will not know whether most normal cell cultures can convert citrulline to arginine. Conversely, more extensive investigations of tumour lines may show just how capable they are, in general, in utilising citrulline (work in progress). The idea that virtually all human melanomas and hepatocellular carcinomas have a similar phenotypic defect with regard to argininosuccinate synthetase is inconsistent with the heterogeneous origins of tumours. Also, this is not true of the mouse melanoma, and leukaemias show the opposite.

Nevertheless, the inability of a number of tumour cell lines to utilise citrulline renders them prime targets for eradication by arginine deprivation, whether achieved by leaving it out in medium formulation or using catabolic enzymes in its presence. This potential Achilles' heel in tumour metabolism deserves further exploitation in view of our previous findings (Scott *et al*, 2000; Wheatley and Campbell, 2001, 2002; Philip *et al*, 2003). Although melanomas do *not exclusively* show this argininosuccinate synthetase deficiency, the findings show that most cases of this dangerous and aggressive tumour can be appropriately and quickly selected for treatment. They are the preferred targets for therapy, for which screening of primary tumours has already begun.
